# SOCIAL DETERMINANTS OF HEALTH ASSOCIATED WITH 30-DAY REHOSPITALIZATION BY USING DISCHARGE SUMMARIES

**DOI:** 10.1093/geroni/igae098.3721

**Published:** 2024-12-31

**Authors:** Youjeong Kang, Jiaying Lu

**Affiliations:** Emory University, Atlanta, Georgia, United States; Emory University, Atlanta, Georgia, United States

## Abstract

**Background:**

There has been increasing interest in the use of Social Determinants of Health (SDOH) in cardiovascular disease outcomes by reducing health disparities. For example, Centers for Medicare and Medicaid Services will require the hospitals reporting five SDOH components (housing, intimate partner violence, utilities, food insecurity and transportation insecurity) in 2025. There is limited evidence which SDOH components are associated with 30-day rehospitalization in HF patients who were in the ICU. Objective: To assess associations between the SDOH and 30-day rehospitalization in HF patients who were in the ICU by using the Medical Information Mart for Intensive Care III (MIMIC-III) data.

**Methods:**

We identified HF patients by using ICD-9 codes related to HF from the MIMIC-III data that is de-identified and is publicly available. We used python to extract SDOH features from discharge summaries and used Chi-square statistical test (whether or not keyword is contained in discharge summaries vs whether or not HF patients have 30-day hospital rehospitalization).

**Results:**

Mean age was 71 (± 14.2), 53% were male, and 73% were white. In particular, keyword “daughter” contributes to the 30-day rehospsitalization. It is unclear how daughter contributes 30-day rehospitalization but “daughter can be one of the SDOH components as social support.

**Conclusion:**

It is difficult to identify how “daughter” is related to 30-day rehospitalization. We will utilize natural language processing models to analyzing the real-world social meaning of SDOH keywords to understand the correlation (e.g. the “daughter” reflecting patients have daughter accompany or other status).

